# [Retracted] Zinc finger protein-like 1 is a novel neuroendocrine biomarker for prostate cancer

**DOI:** 10.3892/ijo.2025.5722

**Published:** 2025-01-20

**Authors:** Neshat Masud, Afaf Aldahish, Kenneth A. Iczkowski, Ajay Kale, Girish V. Shah

Int J Oncol 62: 38, 2023; DOI: 10.3892/ijo.2023.5486

Following the publication of this paper, a concerned reader drew to the attention of the Editorial Office that, for the photomicrographs shown in Fig. 2B on p. 8, the same image had apparently been selected to represent the images for cerebrum and cerebellum from the brain. Secondly, the same image for the prostate tissue in Fig. 2B was used as the 'Matched normal' data in Fig. 3C on p. 9, and thirdly, the si3 and si3+CT images selected for Fig. 7B on p. 13 appeared to be derived from the same original source. Finally, after having performed an independent analysis of the data in the Editorial Office, it was noted that, for the wound healing assay data shown in Fig. 8E on p. 14, overlapping sections were identified comparing the CT and Overexp panels in the lower panel of images in this figure, such that data which were intended to show the results of differently performed experiments had apparently been derived from the same original source.

After having considered these issues, owing to the number of instances of apparently misassembled data that have been identified in this paper, and also on the basis of an overall lack of confidence in the presented data, the Editor of *International Journal of Oncology* has decided that this paper should be retracted from the Journal. The authors were asked for an explanation to account for these concerns, but the Editorial Office did not receive a reply. The Editor apologizes to the readership for any inconvenience caused.

